# Social disparities in access and quality of consultation in outpatient care in Germany

**DOI:** 10.1186/s12875-024-02552-9

**Published:** 2024-08-14

**Authors:** Olaf von dem Knesebeck, Daniel Lüdecke, Jens Klein

**Affiliations:** Institute of Medical Sociology, University Medical Center Hamburg-Eppendorf, Martinistr. 52, 20246 Hamburg, Germany

**Keywords:** Social determinants, Disparities, Primary care, Specialist care, Access, Process quality, Germany

## Abstract

**Background:**

Overall, research on social determinants of access and quality of outpatient care in Germany is scarce. Therefore, social disparities (according to sex, age, income, migration background, and health insurance) in perceived access and quality of consultation in outpatient care (primary care physicians and specialists) in Germany were explored in this study.

**Methods:**

Analyses made use of a cross-sectional online survey. An adult population sample was randomly drawn from a panel which was recruited offline (*N* = 2,201). Perceived access was assessed by waiting time for an appointment (in days) and travel time to the practice (in minutes), while quality of consultation was measured by consultation time (in minutes) and quality of communication (scale of four items, Cronbach’s Alpha 0.89).

**Results:**

In terms of primary care, perceived access and quality of consultation was worse among women compared to men. Estimated consultation time was shorter among people with statutory health insurance compared to privately insured respondents. Regarding specialist care, people aged 60 years and older reported shorter waiting times and better quality of communication. Lower income groups reported lower quality of communication, while perceived access and quality of consultation was worse among respondents with a statutory health insurance. Variances explained by the social characteristics ranged between 1% and 4% for perceived access and between 3% and 7% for quality of consultation.

**Conclusion:**

We found social disparities in perceived access and quality of consultation in outpatient care in Germany. Such disparities in access may indicate structural discrimination, while disparities in quality of consultation may point to interpersonal discrimination in health care.

**Supplementary Information:**

The online version contains supplementary material available at 10.1186/s12875-024-02552-9.

## Background

Social variations in access to health care as well as in utilization and quality of health care have been found in different countries and health care systems [1]. This holds true for different social characteristics like age, sex, migration background, income, and education [2, 3]. In Germany, also health insurance can be regarded as a socioeconomic indicator, because there is a dual structure of statutory and private health insurance [4, 5]. Only people with an income over a certain limit (in 2023: 66,600 Euro per year), self-employed, and public servants can choose a private insurance. Accordingly, about 11% of the German population is privately insured [6].

Reviews on health care inequalities in Germany indicate that there is a particular lack of studies regarding access and quality of outpatient care [4, 7]. Access to health care is considered a multidimensional concept describing the fit between the patient and the health care system [8, 9]. Accessibility (e.g. measured by travel time or distance to the doctor’s practice) and accommodation or adequacy (e.g. measured by waiting time for an appointment) are important dimensions of access. In terms of the latter, a recent international study using data from ten OECD countries [10], explored social variations in waiting times for a general practitioner (GP) appointment and found a negative association with income in four countries (including Germany). Moreover, in three countries, coverage by private health insurance was negatively associated with waiting times for primary care (also including Germany) while differences concerning sex and age were inconsistent. Similar results were found in national studies conducted in Germany [5, 11]. Most German studies on social disparities in waiting times for outpatient care focused on GPs. One study found differences in the magnitude of inequalities between GPs and outpatient specialists [11]. Furthermore, there is a lack of studies considering differences in waiting times according to migration background. In terms of spatial accessibility of health care services, there is evidence for social variations in travel time and distance e.g. from China [12, 13] while there is not much known about the situation in Germany. Greiner et al. [14] found no consistent associations of age, sex, health insurance, and income with walking distance to a GP using data of the German Socio-economic Panel, whereas people with a migration background had a shorter walking distance. However, this study did not consider other travel options.

Studies exploring social differences in the quality of health care in many cases focused on health outcomes like mortality or quality of life [4]. Interpretation of respective findings is impeded as it is often not clear whether they actually reflect disparities in health care since such health outcomes are affected by many other factors [1]. Therefore, studies are needed that consider process indicators like consultation time or quality of patient-provider interaction. It has been shown that longer consultations are of higher quality and are linked to better patient outcomes [15, 16]. Only few studies explored social variations in length of consultations. A British study found that longer consultations were given to older patients while length was not related to patients’ sex or ethnic group [17]. Data from Slovenia indicated that longer consultation time was associated with female gender, higher age, and higher level of education [18]. In terms of quality of patient-provider communication, reviews suggest a social gradient indicating that doctors give more information, more explanations, more (emotional) support and adapt more often a shared decision making style when they meet patients with a higher socioeconomic status [19–21]. However, only very few of the included studies came from Germany: In a study among chronically ill individuals, high income was consistently though weakly associated with perceived quality of doctor-patient relationship [22]. Another German study indicated lower quality of interaction with ambulatory care physicians among patients with statutory health insurance compared to privately insured patients, while differences according to education were inconclusive [23].

Overall, research on social determinants of access and quality of outpatient care in Germany is scarce. Therefore, the general aim of this study was to analyse social disparities in health care in Germany covering different aspects of access and quality of consultation. More specifically, social disparities (according to sex, age, income, migration background, and health insurance) in perceived access (waiting time and travel time) and quality of consultation (consultation time and quality of communication) in outpatient care (primary care physicians and specialists) in Germany were explored.

## Methods

### Study design and sample

Analyses made use of a cross-sectional online survey on social inequalities in health care that was conducted by a social research institute in November and December 2022 (for an excerpt from the questionnaire developed for this study please see supplementary file). An adult population sample (age 18 + years) was randomly drawn from a panel which was recruited offline via telephone. To this end, a dual-frame approach was used that included landline as well as mobile phone numbers. The panel is a population-based, representative sample of the adult population living in Germany. It is regularly refreshed and currently comprises about 120,000 people. Participants are surveyed regularly on different topics. A sample of 5,619 individuals who reported to use the internet was randomly selected from the panel and invited to participate in the present survey via email. After three reminders, *N* = 2,201 individuals participated. We expected about 11% of the respondents to be privately insured. As we also wanted to examine health care variations according to health insurance, we aimed at a sample size of about 2,200. Sample was weighted by age, sex, federal state, and education [24] according to the official statistics of Germany [25]. Therefore, the sample adequately represents the adult population in Germany regarding these socio-demographic characteristics. The survey was approved by the Local Psychological Ethics Committee at the Center for Psychosocial Medicine, University Medical Center Hamburg (No. LPEK-0563).

### Measures

In the survey (lasting 20 min and covering 118 items), various issues of health care inequalities (i.e. access, utilization, quality of care, unmet need, discrimination, and health literacy) were assessed. As for the present analyses and regarding primary care, respondents were asked whether they have a family doctor (yes/no). Analyses on inequalities in primary care were based on the subsample of the respondents who affirmed to have a family doctor (95.2%, *n* = 2,095). With regard to specialist care, it was asked if they visited a specialist at least two times in the last 12 months (yes/no). Analyses on inequalities in specialist care were based on the subsample of the respondents who answered “yes” (55.5%, *n* = 1,221). These respondents were subsequently asked which specialist(s) they visited. Among the specialists were ophthalmologists, gynaecologists, internal specialists, orthopaedists, dermatologists, neurologists, psychiatrists, otolaryngologists, surgeons, urologists, and others. Due to the small number of cases per specialist, they were combined for the analyses. If more than one specialist was mentioned, one of them was randomly selected by the survey tool and the following questions referred to this specialist.

Perceived access and quality of consultation were assessed by two indicators each. In terms of access, respondents were asked to estimate the travel time (open ended in minutes) to the practice of their family doctor (primary care physician) and to the mentioned specialist. Moreover, they were asked to estimate the usual waiting time (open ended in days) to get an appointment with their primary care physician and the specialist. Regarding perceived quality of consultation, respondents should estimate the average consultation time (open ended in minutes) at their primary care physicians and the specialist. Quality of communication was assessed by four items selected from previous studies [26, 27]: “The doctor’s explanations are always understandable to me.” “I have the feeling that the doctor understands me.” “The doctor informs me about my health issues in detail.” “I can talk easily with the doctor.” Responses were given on a four point scale ranging from “completely disagree” (1) to “completely agree” (4). After a principal component analysis indicating a one factor solution, the four items were summarized in a sum scale (Cronbach’s alpha 0.89) divided by the number of items with higher values indicating better perceived quality of communication with the primary care physician and the specialist (range 1–4).

Income, migration background, age, sex, and health insurance were included as social characteristics. Monthly net household income (in Euro) was equalized to consider household size and composition. The variable was divided into quartiles. As for migration background, respondents were categorized into three groups: people who have immigrated themselves (1st generation migrants); people who were born in Germany but whose parents (one of them or both) have immigrated (2nd generation migrants), and those without a migration background. Age was divided into three groups (18–40, 41–59, and 60 + years). Finally, respondents were asked whether they have a private or a statutory health insurance. An excerpt from the questionnaire including all measures described in this paragraph can be found in the supplementary file.

### Analyses

Descriptive statistics (means and standard deviations) will be shown for the four indicators of perceived access and quality of consultation in outpatient care (primary care physicians and specialists). To analyse social disparities in perceived access and quality of consultation, non-parametric tests (Mann Whitney, Kruskal Wallis) were performed for primary care physicians and specialists separately. Moreover, multiple linear regression analyses were conducted, in which all social indicators were introduced simultaneously. Unstandardized regression coefficients, 95% confidence intervals and significances are displayed. The four dependent variables were logarithmised as they were all right-skewed and the regression coefficients were exponentiated. Thus, the coefficients indicate the proportional change in the dependent variable for the related social predictors [28]. Since we assumed that perceived access and quality of consultation vary among the different specialists, we calculated multi-level models for the respective regression analyses. To this end, the speciality was used as a higher-level random effect to account for possible variations, resulting in a nested data structure, because each participant was only assigned to one specialist for the questions on access and quality. For mixed models, variance components (standard deviation (SD) of random effects), conditional and marginal R^2^ are reported. The marginal R^2^ only takes fixed effects into account and is comparable to the R^2^ of the simple linear models. The conditional R^2^ also considers the variation of the higher-level random effects and thus can be interpreted as how much variance in the dependent variable is explained by the full model (including speciality) [29]. As with the coefficients, we have also exponentiated the SD of the random effects so that they are easier to interpret. They show by which factor the mean value of the dependent variable for each specialty group increases or decreases from the dependent variable’s global mean. Hence, the SD indicates the amount of variation for each dependent variable by specialty groups. The significance level for *p*-values was set at *p* < 0.05. Statistical procedures were performed with the statistical program package R 4.3 [30].

**Table 1:** Perceived access and quality of consultation in primary and specialist outpatient care in Germany: means, (standard deviations)

	Access		Quality of consultation	
	Estimated travel time (minutes)	Estimated waiting time (days)	Estimated consultation time (minutes)	Quality of communication (scale, 1–4)
Primary care physicians (*n* = 2,095)	13.6 (10.3)	3.1 (4.8)	12.8 (6.3)	3.4 (0.6)
Specialists (*n* = 1,221)	26.2 (18.7)	30.9 (36.3)	15.0 (10.6)	3.4 (0.7)

## Results

Means and standard deviations of the four indicators of perceived access and quality of consultation in outpatient care (primary care physicians and specialists) are shown in Table 1. In terms of primary care physicians, mean estimated travel time to the practice was 13.6 min and respondents reported that they usually have to wait about three days to get an appointment with their family doctor. Reported consultation time on average was about 13 min. Quality of communication was rated fairly high (mean 3.4). Estimated travel, waiting, and consultation time was considerably higher among specialists (consulted at least two times in the last 12 months) compared to primary care physicians while mean score indicating quality of communication was the same.

**Table 2:** Inequalities in perceived access and quality of consultation (*primary care physicians*): means, (standard deviations), significances (p^a^)

	Access		Quality of consultation	
	Estimated travel time (minutes)	Estimated waiting time (days)	Estimated consultation time (minutes)	Quality of communication (scale, 1–4)
Sex				
female (*n* = 1,071) male (*n* = 1,024) *p*	14.2 (9.9) 13.0 (10.6) < 0.001	3.6 (5.2) 2.6 (4.3) < 0.001	12.5 (6.1) 13.2 (6.6) 0.038	3.4 (0.6) 3.5 (0.5) 0.016
Age				
18–40 years (*n* = 686) 41–59 years (*n* = 706) 60 + years (*n* = 703) *p*	13.6 (11.3) 13.1 (9.8) 14.1 (9.8) 0.010	2.6 (4.0) 3.0 (5.0) 3.8 (5.2) < 0.001	12.6 (6.6) 12.3 (6.1) 13.6 (6.3) < 0.001	3.3 (0.6) 3.4 (0.5) 3.6 (0.5) < 0.001
Income				
1st quartile (*n* = 471) 2nd quartile (*n* = 466) 3rd quartile (*n* = 481) 4th quartile (*n* = 456) *p*	12.8 (9.8) 13.3 (9.7) 14.1 (9.9) 14.2 (11.8) 0.083	3.1 (5.1) 2.8 (3.9) 3.5 (5.2) 2.9 (5.0) 0.335	12.8 (6.5) 12.5 (5.9) 12.9 (6.0) 13.6 (7.0) 0.349	3.5 (0.5) 3.5 (0.6) 3.5 (0.5) 3.4 (0.6) 0.202
Migration background				
no (*n* = 1,617) 1st generation (*n* = 153) 2nd generation (*n* = 324) *p*	13.5 (10.0) 13.8 (10.6) 14.2 (11.6) 0.976	3.1 (4.9) 3.0 (4.3) 3.0 (4.2) 0.730	12.8 (6.2) 12.7 (6.8) 13.1 (7.0) 0.738	3.5 (0.5) 3.4 (0.6) 3.3 (0.6) 0.090
Health insurance				
private (*n* = 260) statutory (*n* = 1,835) *p*	12.5 (8.6) 13.7 (10.4) 0.158	2.8 (3.7) 3.2 (5.0) 0.950	14.6 (7.3) 12.6 (6.2) 0.002	3.5 (0.6) 3.3 (0.6) 0.008

^a^ significance of non-parametric tests (Mann Whitney, Kruskal Wallis)

Case numbers (n) vary due to missing values

Sex and age were significantly associated with perceived access and quality of consultation in primary care (Table 2). Moreover, quality of consultation was rated more favourably by privately insured respondents than by respondents with statutory health insurance. In terms of specialist care (Table 3), there were age differences in waiting and consultation time as well as perceived quality of communication. Quality of communication was rated highest by respondents of the first income quartile. People with statutory health insurance reported longer waiting times, shorter consultation times and less communication quality compared to those who were privately insured.

**Table 3:** Inequalities in perceived access and quality of consultation (*specialists*): means, (standard deviations), significances (p^a^)

	Access		Quality of consultation	
	Estimated travel time (minutes)	Estimated waiting time (days)	Estimated consultation time (minutes)	Quality of communication (scale, 1–4)
Sex				
female (*n* = 697) male (*n* = 524) p	25.8 (18.8) 26.7 (18.5) 0.271	32.3 (35.6) 29.2 (37.2) 0.110	14.5 (10.0) 15.7 (11.3) 0.214	3.3 (0.7) 3.4 (0.6) 0.324
Age				
18–40 years (*n* = 322) 41–59 years (*n* = 405) 60 + years (*n* = 494) p	27.0 (21.7) 25.8 (18.8) 26.0 (16.3) 0.468	31.9 (36.8) 34.7 (38.4) 27.5 (34.0) 0.016	15.8 (11.4) 14.6 (11.6) 14.8 (9.2) 0.028	3.3 (0.7) 3.3 (0.6) 3.6 (0.7) < 0.001
Income				
1st quartile (*n* = 255) 2nd quartile (*n* = 279) 3rd quartile (*n* = 259) 4th quartile (*n* = 255) p	25.8 (18.1) 27.0 (19.4) 25.4 (19.6) 26.4 (19.2) 0.660	31.8 (36.6) 27.7 (36.3) 33.3 (37.8) 31.0 (34.5) 0.240	15.4 (10.3) 14.4 (11.0) 15.4 (10.8) 14.8 (10.1) 0.398	3.5 (0.6) 3.2 (0.7) 3.4 (0.7) 3.2 (0.7) < 0.001
Migration background				
no (*n* = 924) 1st generation (*n* = 103) 2nd generation (*n* = 175) p	26.2 (17.6) 28.3 (25.2) 25.7 (20.2) 0.557	30.8 (35.8) 30.6 (32.5) 32.8 (41.9) 0.934	15.1 (10.3) 14.6 (11.9) 14.9 (11.8) 0.237	3.4 (0.6) 3.2 (0.8) 3.3 (0.6) 0.346
Health insurance				
private (*n* = 139) statutory (*n* = 1,082) p	26.4 (17.4) 26.1 (18.8) 0.582	19.8 (28.9) 32.4 (36.9) < 0.001	19.3 (12.1) 14.5 (10.3) < 0.001	3.7 (0.6) 3.3 (0.7) < 0.001

^a^ significance of non-parametric tests (Mann Whitney, Kruskal Wallis)

Case numbers (n) vary due to missing values

Multiple regression analyses revealed that estimated travel time to primary care practices was significantly higher among females, older age groups, and lower income groups (Table 4). For example, travel time among females was 13% longer (regression coefficient 1.13) than among males. Estimated waiting time was also longer among women and higher age groups. Consultation time was positively associated with old age and low income while it was negatively related to statutory insurance. Quality of communication was negatively associated with female sex as well as migration background, and positively related to age. Explained variances ranged between 2% and 7%.

**Table 4:** Multiple regression analyses (*primary care physicians*): unstandardized regression coefficients, (95% confidence intervals), significances (*N* = 2,095)

	Access		Quality of consultation	
	Estimated travel time	Estimated waiting time	Estimated consultation time	Quality of communication
Sex				
female	1.13 (1.06–1.20)***	1.24 (1.13–1.35)***	0.96 (0.92–1.01)	0.97 (0.95–0.98)***
Age				
41–59 years 60 + years	1.03 (0.95–1.12) 1.14 (1.05–1.24)**	1.09 (0.98–1.21) 1.25 (1.12–1.39)***	1.00 (0.94–1.06) 1.12 (1.06–1.19)***	1.04 (1.02–1.06)*** 1.10 (1.08–1.13)***
Income				
2nd quartile 3rd quartile 4th quartile	1.03 (0.94–1.13) 1.12 (1.02–1.23)* 1.07 (0.98–1.18)	0.99 (0.87–1.12) 1.09 (0.97–1.23) 0.97 (0.85–1.10)	1.00 (0.93–1.06) 1.05 (0.98–1.12) 1.10 (1.03–1.18)**	0.98 (0.96-1.00) 1.00 (0.97–1.02) 0.98 (0.96-1.00)
Migration background				
1st generation 2nd generation	0.96 (0.84–1.09) 1.00 (0.91–1.10)	1.04 (0.88–1.24) 1.08 (0.96–1.22)	0.97 (0.88–1.06) 1.01 (0.94–1.08)	0.96 (0.93-1.00)* 0.97 (0.94–0.99)**
Health insurance				
statutory	1.06 (0.95–1.17)	1.00 (0.87–1.14)	0.85 (0.79–0.91)***	0.98 (0.96–1.01)
R^2^	0.02	0.03	0.03	0.07

Reference categories: male, 18–40 years, 1st income quartile, no migration background, private health insurance

* *p* < 0.05, ** *p* < 0.01, *** *p* < 0.001

Regarding specialist care, there were no significant associations of the social characteristics with estimated travel time according to the multiple regression analyses (mixed models, Table 5). Waiting time was estimated to be shorter among old respondents and longer among publicly insured individuals. The latter had a 62% longer waiting time than respondents with a private health insurance (regression coefficient 1.62). Those with a statutory insurance also reported significantly shorter consultation times. Finally, there were significant associations of perceived communication quality with age, income, migration background, and health insurance. Marginal R^2^ varied between 1% and 6%, while the conditional R^2^ varied between 6% and 13%. Looking at the random effects, we found the highest variation between specialist types for the estimated waiting time (on average 37% lower or higher from mean estimated waiting time, SD 1.37), while the quality of communication hardly varied (SD 1.03).

**Table 5:** Multiple regression analyses (mixed models, *specialists*): unstandardized regression coefficients, (95% confidence intervals), significances (*N* = 1,221)

	Access		Quality of consultation	
	Estimated travel time	Estimated waiting time	Estimated consultation time	Quality of communication
Sex				
female	1.02 (0.93–1.12)	1.09 (0.92–1.29)	0.92 (0.85-1.00)	0.98 (0.95–1.01)
Age				
41–59 years 60 + years	0.99 (0.89–1.11) 1.03 (0.92–1.16)	1.12 (0.91–1.38) 0.79 (0.64–0.97)*	0.92 (0.83–1.01) 1.03 (0.93–1.13)	1.02 (0.99–1.06) 1.09 (1.05–1.13)***
Income				
2nd quartile	1.03 (0.92–1.16)	0.81 (0.65–1.01)	0.95 (0.86–1.05)	0.93 (0.90–0.97)***
3rd quartile	0.95 (0.85–1.07)	0.96 (0.77–1.21)	1.01 (0.91–1.12)	0.96 (0.92–0.99)*
4th quartile	1.02 (0.90–1.15)	0.88 (0.70–1.11)	0.99 (0.89–1.11)	0.94 (0.90–0.98)**
Migration background				
1st generation	0.98 (0.85–1.14)	1.05 (0.79–1.38)	0.89 (0.78–1.02)	0.94 (0.89–0.99)*
2nd generation	0.98 (0.87–1.10)	0.98 (0.79–1.22)	0.94 (0.85–1.04)	0.99 (0.96–1.03)
Health insurance				
statutory	0.95 (0.84–1.09)	1.62 (1.26–2.07)***	0.76 (0.68–0.86)***	0.94 (0.90–0.98)**
Random effects				
SD (Intercept: specialty)	1.19	1.37	1.21	1.03
Conditional R^2^	0.06	0.10	0.13	0.07
Marginal R^2^	0.01	0.04	0.04	0.06

Reference categories: male, 18–40 years, 1st income quartile, no migration background, private health insurance

* *p* < 0.05, ** *p* < 0.01, *** *p* < 0.001; SD standard deviation

## Discussion

### Summary

In this study, social disparities (according to sex, age, income, migration background, and health insurance) in perceived access and quality of consultation in outpatient care were analysed. In terms of primary care, perceived access and quality of consultation was worse among women compared to men. Travel and waiting time was longer among people aged 60 years and older but quality of consultation was better. Income and migration background were weakly and inconsistently associated with access and quality of consultation. Estimated consultation time was shorter among people with statutory health insurance. Regarding specialist care, sex and migration background were weakly and inconsistently related to perceived access and quality of consultation. People aged 60 years and older reported shorter waiting times and better quality of communication. Lower income groups reported lower quality of communication compared to the highest income quartile. Finally, perceived access and quality of consultation was worse among respondents with a statutory health insurance compared to privately insured respondents. Overall, variances explained by the social characteristics were fairly low (ranging between 1% and 7%).

### Interpretation

Access to health care is a multidimensional concept dealing with the fit between patient and the health care system [8, 9]. In our study, we used indicators for two dimensions: accessibility (travel time to the doctor’s practice) and adequacy (waiting time for an appointment). Regarding accessibility of primary care, women and older people reported longer travel times while there were no consistent associations with income, migration, and health insurance, and we did not find any inequalities in accessibility of specialist care. This is noteworthy as there is a discussion about maldistribution of outpatient care to the disadvantage of deprived areas and individuals in Germany [14, 31]. However, respective empirical studies are scarce and our results are basically in line with those of a previous German study that analysed walking distance to a GP [14]. Waiting time to get an appointment was reported to be longer among women and older people in case of a primary care physician while again there were no consistent associations with income, migration, and health insurance. Differences according to sex and age may be associated with increased utilisation of primary care among women and older people [32]. In terms of age differences, one should additionally keep in mind that short-term appointments among older people may be less critical as they are more often affected by chronic conditions which are planned and managed continuously. Our results on waiting times in primary care partly differ from previous studies [5, 10, 11] which can be explained by time of data collection and different measurements of waiting times.

Regarding specialist care, one predictor stood out in our analyses: People with a statutory health insurance waited significantly longer than privately insured individuals. This obviously has not changed in the last 15 years as type of health insurance was also the strongest predictor for waiting time for an appointment with the specialist in a study using data from 2009 [11]. The persistence of this inequality in access to specialist care is remarkable because appointment service centres were established in 2019 by the National Association of Statutory Health Insurance Physicians in Germany to promote faster appointments for publicly insured patients [33]. This measure obviously was not successful in reducing inequalities in waiting times. For an explanation, it has to be kept in mind that differences in physician reimbursement rates create incentives for the preferential treatment of privately insured patients in the outpatient setting in Germany. Physicians get even lower reimbursement rates for publicly insured patients when they exceed a certain amount of health services per quarter because there is a budgeting of outpatient care. Moreover, there is an increasing shortage of doctors in Germany, especially in rural areas. It seems manifest that the combination of this reimbursement policy and a shortage of supply contributes to the persistence of disparities in waiting times according to health insurance.

Access to health care is a core component of universal health coverage. Barriers arising from the accessibility and adequacy can lead to unmet need due to distance and waiting times [34]. There is also concern that long waiting times may worsen health outcomes [35].

Perceived quality of consultation was assessed by two indicators: estimated consultation time and quality of communication (sum scale of four items). In terms of the former, respondents with a statutory health insurance reported significantly shorter consultation times in primary and specialist care than those with a private health insurance. These inequalities are probably also due to the differences in the reimbursement system discriminating statutorily insured patients. Regarding primary care, people 60 years and older and those from the lowest income groups reported longer consultation times. The few previous studies (which were not conducted in Germany) also found associations between longer consultations and higher age [17, 18] which may indicate the increased effort for the management of older patients often affected by multimorbidity [36]. Higher age was also associated with better perceived quality of communication in primary and specialist care in our study. Quality of communication was worse among individuals with migration background (primary care physicians and specialists) as well as among those with lower income and a statutory health insurance (specialists). Socioeconomic inequalities in doctor-patient communication have also been found in other countries [19–21]. However, there was not much known about the situation in Germany. In our study, income-related disparities in quality of communication were restricted to specialist care just as differences according to health insurance. This is in line with a previous German study showing a lower quality of interaction with specialists among patients with statutory health insurance compared to privately insured patients [23].

The finding that people with a statutory health insurance reported significantly shorter consultation times is relevant as it has been shown that longer consultations are of higher quality and are linked to better patient outcomes [15, 16]. It has also been shown that the quality of patient-provider communication can predict health and well-being [37]. Thus, social disparities in quality of consultation may contribute to health inequalities.

### Limitations

Our findings have to be interpreted against the background of some limitations. Analyses made use of an online survey. Even though a random sample was drawn from a panel which was recruited offline, only individuals who use the internet were included. This may lead to a selection bias, just as the fact that only about 39.2% of the invited persons participated. To reduce this potential bias, data was weighted by age, sex, federal state, and education according to the official statistics using an iterative proportional fitting approach [24]. Moreover, analyses only included individuals who were able to read German. This has to be considered when interpreting results on variations according to migration history. Due to the sample size, we combined the different specialists for the analyses which in a way is crude. To consider variations between the different specialists, mixed models were applied in the regression analyses. Furthermore, we only used self-reported measures for access and quality of consultations. For three indicators, respondents were asked to estimate time (travel, waiting, and consultation time). Although previous studies also used self-reports [5, 10, 14, 17], such estimations do not necessarily reflect the real lengths of time. For consultation time, one study showed that patients tend to overestimate duration [38], while another indicated an underestimation [17]. Also, the scale on quality of communication was not previously validated. However, the four items used were taken from previous studies [26, 27] and internal consistency was good (Cronbach’s alpha 0.89). Finally, the relevance of some statistically significant social differences in access and quality is difficult to evaluate as there are no validated thresholds. There are studies suggesting that a change of approximately half a standard deviation in the outcome can be considered as a minimal important difference, almost independent of the scale [39]. However, as this suggestion is related to studies investigating health-related quality of life, transfer to our indicators is questionable.

## Conclusions

This is one of the first studies, analysing social disparities in perceived access and quality of consultation in outpatient care (primary care physicians and specialists) in Germany. Social disparities in access may indicate structural discrimination, while disparities in quality of consultation may point to interpersonal discrimination in health care [40]. Thus, measures to reduce inequalities should comprise interventions targeting the structural and the interpersonal level. In terms of the former, abolition of the dual structure of statutory and private health insurance would certainly reduce disparities in health care. This can be expected particularly for specialist care where differences in waiting times according to health insurance were found to be consistent and distinct in our study. Accordingly, an abolition of the dual structure could also reduce unmet need among privately insured people. Disparities in the quality of communication may be reduced by increasing doctor’s awareness of social differences in communicative behaviour and information needs and by empowering patients to express concerns and preferences [20]. Awareness of social differences should be addressed in medical education and in continuing medical training, while empowerment of patients can be realized by increasing health literacy [41] and by promoting patient activation to support shared decision-making [42]. Such interventions may also improve adherence, self-care skills, and well-being among deprived social groups [37].

### Electronic supplementary material

Below is the link to the electronic supplementary material.

Supplementary Material 1

## Data Availability

The dataset used is available from the corresponding author on reasonable request.

## Abbreviations

GP: General practitioner
