# Supplementary material for: Social disparities in access and quality of consultation in outpatient care in Germany
Source: BMC Prim Care. 2024 Aug 14;25:299. doi: 10.1186/s12875-024-02552-9 (PMC11323346; doi:10.1186/s12875-024-02552-9)
Supplement: Supplementary file 1 — Supplementary Material 1 [file 12875_2024_2552_MOESM1_ESM.docx]

**Excerpt from the questionnaire**

|  | Do you have a family doctor? | | | |
| --- | --- | --- | --- | --- |
|  | ○ yes | ○ no (filter) | | *+don’t know/no answer* |
|  | Please estimate the travel time to the practice of your family doctor (in minutes). | | | |
|  | _________ minutes | | ○ don’t know | |
|  | Please estimate the usual waiting time to get an appointment with your family doctor (in days). | | | |
|  | _________ days | | ○ don’t know | |
|  | Please estimate the average consultation time at your family doctor (in minutes) | | | |
|  | __________ minutes | | ○ don’t know | |

|  | Please indicate whether you agree to the following statements regarding your family doctor. | | | | | | |
| --- | --- | --- | --- | --- | --- | --- | --- |
|  |  | Completely agree | | Rather agree | Rather disagree | Completely disagree | *Don’t know* |
|  | The doctor’s explanations are always understandable to me. | ○ | | ○ | ○ | ○ | ○ |
|  | I have the feeling that the doctor understands me. | ○ | | ○ | ○ | ○ | ○ |
|  | The doctor informs me about my health issues in detail. | | ○ | ○ | ○ | ○ | ○ |
|  | I can talk easily with the doctor. | | ○ | ○ | ○ | ○ | ○ |

|  | Did you visit a specialist at least two times in the last 12 months? | | |
| --- | --- | --- | --- |
|  | ○ yes | ○ no (filter) | ○ don’t know |
|  | Which specialist did you visit? | | |
|  | __________________ | | ○ don’t know |
|  | The following questions refer to this specialist.  Please estimate the travel time to the practice of this specialist (in minutes). | | |
|  | _________ minutes | | ○ don’t know |
|  | Please estimate the usual waiting time to get an appointment with the specialist (in days). | | |
|  | _________ days | | ○ don’t know |
|  | Please estimate the average consultation time at this specialist (in minutes) | | |
|  | __________ minutes | | ○ don’t know |

|  | Please indicate whether you agree to the following statements regarding this specialist. | | | | | | |
| --- | --- | --- | --- | --- | --- | --- | --- |
|  |  | Completely agree | | Rather agree | Rather disagree | Completely disagree | *Don’t know* |
|  | The doctor’s explanations are always understandable to me. | ○ | | ○ | ○ | ○ | ○ |
|  | I have the feeling that the doctor understands me. | ○ | | ○ | ○ | ○ | ○ |
|  | The doctor informs me about my health issues in detail. | | ○ | ○ | ○ | ○ | ○ |
|  | I can talk easily with the doctor. | | ○ | ○ | ○ | ○ | ○ |

|  | What is your gender? | | |
| --- | --- | --- | --- |
|  | ○ male | ○ female | ○ diverse |
|  | How old are you? | | ________ years |

|  | In which country were you born? | | | |
| --- | --- | --- | --- | --- |
|  | ○ in Germany | | ○ other country: _________________ *+ no answer* | |
|  | In which country was your mother/father born? | | | |
|  | Your mother: | ○ Germany | | ○ other country: ________________ |
|  | Your father: | ○ Germany | | ○ other country: ________________ |

| What is your net household income per month? |
| --- |
| ○ less than 1,000 € |
| ○ 1.000 € - 1,499 € |
| ○ 1.500 € 1,999 € |
| ○ 2.000 € - 2,499 € |
| ○ 2.500 € - 2,999 € |
| ○ 3.000 € - 3,499 € |
| ○ 3.5000 € 3,999 € |
| ○ 4,000 € - 4,999 € |
| ○ 5,000 € - 5,499 € |
| ○ 5,500 € - 5,999 € |
| ○ 6,000 € or more |
| ○ don’t know/no answer |

|  | Do you have a private or a statutory health insurance? | | | | | |
| --- | --- | --- | --- | --- | --- | --- |
|  | ○ private | ○ statutory | ○ don’t know |  |  |  |
